# Genetic characteristics and antimicrobial resistance of Staphylococcus epidermidis isolates from patients with catheter-related bloodstream infections and from colonized healthcare workers in a Belgian hospital

**DOI:** 10.1186/1476-0711-13-20

**Published:** 2014-06-04

**Authors:** Soraya Cherifi, Baudouin Byl, Ariane Deplano, Carole Nagant, Claire Nonhoff, Olivier Denis, Marie Hallin

**Affiliations:** 1 Infection Control Unit, Brugmann University Hospital, 4 Place Van Gehuchten, 1020 Brussels, Belgium; 2 Infection Control Unit, Erasme University Hospital, 808 Route de Lennik, 1070 Brussels, Belgium; 3 School of Public Health, Université Libre de Bruxelles (ULB), Brussels, Belgium; 4 Centre National de Référence Staphylococcus aureus, Microbiology Department, Erasme University Hospital, ULB, 808 Route de Lennik, 1070 Brussels, Belgium; 5 Laboratoire de Chimie biologique et médicale et de Microbiologie pharmaceutique, Faculté de Pharmacie, ULB, CP 205/02 Boulevard du Triomphe, 1050 Brussels, Belgium; 6 Centre de Diagnostic Moléculaire, iris-Lab, 322 Rue Haute, 1000 Brussels, Belgium

**Keywords:** *Staphylococcus epidermidis*, Catheter-related bloodstream infection, Molecular epidemiology, PFGE, MLST

## Abstract

**Background:**

*Staphylococcus epidermidis* is a pathogen that is frequently encountered in the hospital environment. Healthcare workers (HCWs) can serve as a reservoir for the transmission of *S. epidermidis* to patients.

**Methods:**

The aim of this study was to compare and identify differences between *S. epidermidis* isolated from 20 patients with catheter-related bloodstream infections (CRBSIs) and from the hands of 42 HCWs in the same hospital in terms of antimicrobial resistance, biofilm production, presence of the intercellular adhesion (*ica)* operon and genetic diversity (pulsed field gel electrophoresis (PFGE), multilocus sequence typing (MLST) and staphylococcal cassette chromosome (SCC) *mec* typing).

**Results:**

*S. epidermidis* isolates that caused CRBSI were resistant to significantly more non-betalactam drugs than were isolates collected from HCWs. Among the 43 *mecA* positive isolates (26 from HCWs), the most frequent SCC*mec* type was type IV (44%). The *ica* operon was significantly more prevalent in CRBSI isolates than in HCWs (*P* < 0.05). Weak *in vitro* biofilm production seemed to correlate with the absence of the *ica* operon regardless of the commensal or pathogenic origin of the isolate. The 62 isolates showed high diversity in their PFGE patterns divided into 37 different types: 19 harbored only by the CRBSI isolates and 6 shared by the clinical and HCW isolates. MLST revealed a total of ten different sequence types (ST). ST2 was limited to CRBSI-specific PFGE types while the “mixed” PFGE types were ST5, ST16, ST88 and ST153.

**Conclusion:**

One third of CRBSI episodes were due to isolates belonging to PFGE types that were also found on the hands of HCWs, suggesting that HCW serve as a reservoir for oxacillin resistance and transmission to patients. However, *S. epidermidis* ST2, *mecA-*positive and *icaA*-positive isolates, which caused the majority of clinically severe CRBSI, were not recovered from the HCW’s hands.

## Introduction

Catheter-related bloodstream infection (CRBSI) is one of the most common healthcare-associated infections [1], and coagulase-negative staphylococci, predominantly *Staphylococcus epidermidis*, are the most common pathogens involved. A key factor in the pathogenesis of *S. epidermidis* CRBSI is its ability to form a biofilm, which is mediated by the production of an intercellular polysaccharide adhesin (PIA) encoded by an accessory gene cluster called the intercellular adhesion (*ica)* operon [2].

In a recent study, the genotypes of 33 *S. epidermidis* isolates from blood cultures of patients with nosocomial CRBSI have been investigated and compared with 33 commensal *S. epidermidis* isolates from healthy students [3]. The two populations were found to be genetically different using pulsed field gel electrophoresis (PFGE), a typing method known to provide reliable short-term epidemiological data: only 23% of the 37 PFGE types observed were common to both CRBSIs and commensal isolates. Furthermore, clinically severe CRBSIs were due to multidrug-resistant *ica* positive isolates. Using multilocus sequence typing (MLST), a method used to study long-term global epidemiology, these isolates were found to belong to two closely related genetic lineages (sequence type (ST)2 and ST54), which were not found among the healthy volunteers (HVs). Similarly, Muldrew et al. demonstrated the presence of a predominant and persistent clone among central line isolates from a specific ward. They showed that this clone was not found among *S. epidermidis* isolates collected from a control group living in the community [4]. These data suggest that particular lineages are transmitted within the hospital, which, by combining resistance and virulence, can cause more severe disease. However, controversy remains over the source of these bacteria isolated from central venous catheters. Cross-transmission of these pathogens might occur directly between patients in contact with one another, indirectly through contamination from the environment or through the contaminated hands of healthcare workers (HCWs). Indeed, acquisition of antibiotic-resistant flora such as methicillin-resistant *S. epidermidis* (MRSE) is an occupational hazard for HCWs. Such flora can serve as a reservoir for transmission to the environment, other staff, and ultimately to patients. Several studies have demonstrated that HCWs carry *S. epidermidis* that belong to the same clones that cause infections in their respective wards [5-7].

In this study we analyzed the antimicrobial drug resistance and molecular epidemiology of *S. epidermidis* that resulted in CRBSIs and commensal *S. epidermidis* collected from HCWs working in the same hospital over a close period.

We also studied the correlation between *in vitro* biofilm formation and the presence of the *ica* operon in this collection and on a set of commensal *S. epidermidis* isolates from HVs and from CRBSIs, which were previously collected [3]. The aim was to compare and identify differences between the *S. epidermidis* isolates causing CRBSI and commensal isolates from HCWs and HVs in terms of genetic relatedness, resistance to antibiotics, presence of the *ica* operon, and *in vitro* biofilm formation.

## Methods

During two days in September 2012, 51 HCWs volunteered to participate in this study. They worked in the ten wards of the Erasme University Hospital that present the majority of reported CRBSIs cases, such as the intensive care department and gastroenterology department.

At least five HCWs per unit were sampled. Samples were obtained by taking fingerprints from the dominant hand directly onto mannitol salt agar plates (Becton-Dickinson, Heidelberg, Germany) at the end of the morning from HCWs that have already worked for several hours. Plates were incubated at 35°C in an aerobic atmosphere for two days, and a maximum of four mannitol non-fermentative colonies were randomly picked and sub-cultured onto Columbia blood agar for 18–24 hours at 35°C in an aerobic atmosphere. Matrix-assisted laser desorption/ionization time-of-flight mass spectrometry (MALDITOF-MS) was used to identify the bacteria present in each specimen. A maximum of one isolate per volunteer (the first one identified as *S. epidermidis*) was selected for the study.

The clinical strains originated from blood cultures of patients with CRBSI and were identified through the Erasme University Hospital Infection Control Unit database from January 2011 to September 2012. Among the 56 CRBSIs episodes identified, 20 episodes due to *S. epidermidis* (at least two blood cultures; one episode per patient) and treated with antibiotics were included. The patients were located on various wards, including the intensive care unit (n = 10), gastroenterology (n = 7), nephrology (n = 1), neurology (n = 1), and thoracic surgery (n = 1) departments. All of the 20 episodes of CRBSI except one were hospital-acquired and were distributed through non-tunneled central venous catheters (n = 16), peripheral arterial catheters (n = 2), peripheral venous catheter (n =1) and tunneled central venous catheter (n = 1).

### Definitions

Confirmation of a *S. epidermidis*-BSI requires that the patient presents with fever (temperature above 38°C) or hypothermia (temperature below 35°C) and/or hypotension and at least two pairs of blood cultures positive for *S. epidermidis* collected at different times. We defined *S. epidermidis-*CRBSI as a primary BSI with a catheter positive for *S. epidermidis* presenting an identical susceptibility profile to the blood cultures. Clinically, the condition was considered severe if the patient presented with severe sepsis or septic shock when blood samples were collected for culture. Multidrug resistance (MDR) was defined as resistance to at least 5 of the 12 antibiotics tested in the study.

### Bacterial isolate identification (ID) and susceptibility testing

Isolates, conserved at −80°C in pure glycerin, were sub-cultured onto Columbia blood agar for 18–24 hours at 35°C in an aerobic atmosphere. ID was confirmed by MALDITOF-MS. Susceptibility to 16 antimicrobial agents (penicillin, cefoxitin -used as marker for the detection of methicillin resistance-, erythromycin, clindamycin, levofloxacin, gentamicin, kanamycin, tobramycin, fusidic acid, minocycline, rifampin, trimethoprim-sulfamethoxazole, mupirocin, vancomycin, linezolid, and tigecycline) was determined by Vitek 2® using the antibiotic susceptibility P-610 card.

Bacterial DNA was extracted as described by Ünal et al. [8], and subsequently all isolates were tested for the presence of the *mecA* gene as previously described [9]. The presence of the antibiotic resistance genes *aadC, aacA-aphD, aph3*, *erm* (A, B and C), and *msrA* was determined using PCR on all kanamycin, tobramycin, gentamycin and erythromycin non-susceptible isolates [10,11]. Additionally, all isolates were tested for the presence of *icaA* to assess the presence of the *ica* operon [12].

### Molecular typing techniques

For all of the isolates, *Sma*I genomic DNA restriction fragments were separated by PFGE as previously described [13]. As established by the previous criteria [14], patterns differing by less than 79% (corresponding to less than seven band differences) were considered to belong to the same type (represented by a letter). Arbitrarily, PFGE types represented by a single isolate were called “singletons”, while those represented by three or more isolates were considered “epidemic types.”

MLST was performed as described by Thomas et al. [15] on a randomly selected isolate from each PFGE type represented by more than one isolate. SCC*mec* typing was performed on all isolates based on determination of the *ccr* and *mec* gene complex using M-PCR1 and M-PCR2 as previously described [16].

### Comparative analysis of the isolates from CRBSIs, HVs and HCWs

We compared a total of 53 CRBSI isolates (20 collected from the present study and 33 collected previously [3]) to commensal *S. epidermidis* isolated from the 42 HCWs from the present study and from the 33 HVs of our previous study [3] in terms of genetic relatedness, resistance to antibiotics, and biofilm formation.

### Study of biofilm formation with the crystal violet staining method on 128 *S. epidermidis* isolates

An overnight growth culture in brain heart infusion (BHI) liquid was adjusted to a final OD_600_ of 1.00 ± 0.05 by adding sterile BHI medium. This OD-adjusted suspension was then diluted 250-fold to obtain the initial bacterial suspension (IBS). The wells of a 96-well microplate were inoculated with 200 μL of the IBS and incubated at 35°C in a humid atmosphere. A control well was inoculated with sterile BHI medium. Each isolate was evaluated using six samples. The medium was removed and the wells were washed three times with sterile distilled water. The wells were air dried for 45 min, and the adherent cells were stained with a 1/1000 solution of crystal violet. After 45 min, the excess crystal violet was removed and the wells were washed five times with 300 μL sterile distilled water. The dye was dissolved with 200 μL of a 33% acetic acid solution, and the absorbance of each well was read at 540 nm in a microplate reader (Synergy HT, BioTek), as described by Stepanovic et al. [17].

### Confidentiality and ethics committee approval

The demographic, clinical, microbiological and molecular epidemiology data were collected anonymously. The ethics committee of the Erasme Hospital approved the protocol before the beginning of the study (No. P2012/208).

### Data analysis

Stata data analysis and statistical software (version 12.0; StataCorp LP: College Station, Texas, USA) were used for all data analyses. Categorical analyses were carried out using Fisher’s exact test or the chi-square test, as appropriate. The Mann–Whitney *U* test or Kruskal-Wallis test was used for comparison between groups of non-normally distributed variables, as appropriate. Statistical significance was set at a two-sided *P-*value of < 0.05.

## Results

A total of 62 *S. epidermidis* isolates (42 from HCWs and 20 from CRBSIs) were collected. All of the isolates were confirmed as *S. epidermidis* by MALDITOF-MS analysis. Overall, *S. epidermidis* was recovered from the hands of 82% of the HCWs (42 out of 51).

### Resistance

The resistance results are shown in Table 1. The majority of isolates (69%) were resistant to methicillin, and there were no significant difference in the presence of the *mecA* gene between the HCW and CRBSI isolates. *S. epidermidis* isolates from patients with CRBSI were significantly more resistant to all non-betalactam drugs, except for erythromycin and fusidic acid, than the *S. epidermidis* isolates collected from HCWs. A higher percentage of mupirocin-resistant strains was found among isolates from CRBSIs in comparison with HCWs (*P = *0.013).

**Table 1:** **Antimicrobial resistance profiles and resistance (R)-encoding genes of *S. epidermidis *** **isolates collected from healthcare workers (HCWs; n =42) vs. *S. epidermidis *** **isolates causing catheter-related bloodstream infections (CRBSIs; n = 20)**

***Antimicrobial***	***Encoding gene***	***HCWs (% R)***	***CRBSIs (% R)***	***P-value***
**Penicillin**		37 (88%)	20 (100%)	0.165
**Methicillin (cefoxitin)**		26 (62%)	17 (85%)	0.065
	*mecA*	*26*	*17*	0.065
**Erythromycin**		26 (62%)	15 (75%)	0.308
	*ermC*	*11*	*12*	0.009
	*msrA*	*13*	*0*	0.006
	*ermA*	*1*	*3*	0.034
**Clindamycin**		14 [6]* (33%)	15 [5]* (75%)	0.002
**Levofloxacin**		10 (24%)	14 (70%)	< 0.001
**Fusidic acid**		19 (45%)	13 (65%)	0.146
**Trimethoprim/sulfamethoxazole**		4 (10%)	11 (55%)	< 0.001
**Aminoglycosides§ (at least one)**		10 (24%)	13 (65%)	0.002
**Kanamycin**		9 (21%)	13 (65%)	< 0.001
**Tobramycin**		6 (14%)	13 (65%)	< 0.001
**Gentamicin**		5 (12%)	10 (50%)	0.003
	*aadC*	*6*	*11*	< 0.001
	*aacA-aphD*	*5*	*10*	0.003
	*aph3*	*3*	*1*	1.000
**Mupirocin**		4 (10%)	8 (40%)	0.013
**Rifampin**		0 (0%)	5 (25%)	0.002
**Median sum of resistance to non-beta-lactams (range)**		2 (0–5)	5 (0–8)	< 0.001

Legend:

No resistance to minocycline, vancomycin, linezolid, and tigecycline was observed.

^§^The three aminoglycosides were regarded as a whole, and isolates resistant to at least one of the three drugs were considered resistant to aminoglycosides.

*For clindamycin, the values in square brackets represent the number of isolates with a clindamycin-inducible resistant phenotype.

When we assayed the genes that encode these resistance profiles, 23 erythromycin-resistant isolates had the *ermC* gene, whereas 13 isolates (only from HCWs) were *msrA* positive. Constitutive phenotypes were harbored by isolates with the *ermC* (n = 13) and *ermA* (n = 3) genes. When present, aminoglycoside resistance was mainly mediated by *aadC* (n = 17) alone or in combination with *aacA-aphD* (n = 10).

#### Comparative resistance data from CRBSI, HCW and HV isolates

A total of 128 isolates were analyzed, classified into three groups: HCWs (n = 42), CRBSIs (n = 53) and HVs (n = 33). Among the 12 antibiotics tested, which included the betalactams, the median sum of resistances observed for HCWs, HVs and CRBSIs were 4, 1, and 6, respectively. The difference in these values was statistically significant (*P* < 0.001). The difference in resistance to methicillin among the three sub-populations was also statistically significant (*P* < 0.001).

### Molecular epidemiology

#### PFGE

The 62 isolates produced a broad range of restriction patterns, which were distributed into 37 different PFGE types (see Table 2). Twenty-three PFGE types were represented by a single isolate (singletons). Eight PFGE types (of which six singletons) were harbored by CRBSI isolates only. Twenty-three PFGE types (17 singletons) were harbored by a total of 34 HCW isolates. Six PFGE types were shared by CRBSI and HCW isolates (n = 17).

**Table 2:** **PFGE types and Sequence types (ST) distribution of *S. epidermidis *** **isolates collected from health care workers (HCW) and catheter-related bloodstream infections (CRBSI)**

		**N° of isolates**	
**PFGE type**	**ST**	**HCW**	**CRBSI**
***Y***	***ST5***	***3***	***3***
*I*	*ST22*	*5*	*0*
*ZB*	*ST33*	*4*	*0*
*ZH*	*ST2*	*0*	*3*
***ZV***	***ST5***	***1***	***2***
R	ST88	2	0
YA	New single locus variant of ST370	2	0
YI	ST16	2	0
ZO	ST130	2	0
ZL	ST2	0	2
**ZU**	**ST5**	**1**	**1**
**F**	**ST153**	**1**	**1**
**YM**	**ST16**	**1**	**1**
**ZR**	**ST88**	**1**	**1**
Singletons	ND	17	6
Total		42	20

Legend:

*ND* not done.

Mixed PFGE types are identified by bold type.

Epidemic PFGE types are identified by italic type.

#### MLST

Fourteen isolates were typed by MLST (one randomly selected per PFGE type composed of more than one isolate). MLST revealed a total of ten different sequence types. ST2 was limited to CRBSI-specific PFGE types while the “mixed” PFGE types were ST5, ST16, ST88 and ST153. HCW-specific PFGE types were ST22, ST33, ST88, ST130 and a new ST single locus variant (SLV) ST370.

#### SCCmec type and presence of the ica operon

Among the 43 *mecA* positive isolates (26 HCWs), nine different combinations of *ccr* and *mec* complexes were found. The most frequent type was SCC*mec* type IV (*n* = 19). Up to 12 isolates carried composite SCC*mec* types, amplifying more than one *ccr* gene complex. Eleven isolates were unable to be SCC*mec* typed because of the absence of amplification for one of the two complexes.

We observed *icaA* positive isolates in both HCWs and patients with a ratio of 50% (10 out of 20) for CRBSI isolates and 24% (10 out of 42) for HCW isolates (*P = *0.04).

#### Comparative molecular epidemiology of CRBSI, HCW and HV isolates

Among the 128 isolates, 64 different PFGE types were found, which included 41 singletons. Sixty-five isolates (51%) belonged to one of the 12 epidemic PFGE types that were identified. Distribution of epidemic *S. epidermidis* PFGE types is shown in Table 3. Four epidemic PFGE types, representing a total of 24 isolates (19%), were found among the three epidemiological groups (group 1). Few group 1 isolates were *mec*A positive, but all were *ica*A negative. Three epidemic PFGE types, representing 20 isolates (16%), were only found in the hospital environment (group 2). All group 2 isolates were *mec*A positive, but only five isolates were *ica*A positive. Three epidemic PFGE types (12 isolates, 9%) were exclusively found among CRBSIs (“CRBSI-specific” PFGE types, group 3). All group 3 isolates were *mec*A and *ica*A positive and possessed MDR. One epidemic PFGE type (five isolates) was found among both HCWs and HVs, and one epidemic PFGE was found (four isolates) among only HCWs (“commensal-specific” epidemic PFGE types, group 4). Both of these group 4 epidemic PFGE types were *mec*A and *ica*A negative.

**Table 3:** **Distribution of epidemic *S. epidermidis *** **PFGE types (≥3 isolates) and related characteristics**

	***Group 1***	***Group 2***	***Group 3***	***Group 4***	***P- value***
	***Common to the three epidemiological groups (CRBSIs-HCWs-HVs)***	***Specific to hospital environment (CRBSIs-HCWs)***	***Specific to CRBSIs***	***Commensal (HCWs, HCWs-HVs)***	
**N° isolates**	24	20	12	9	NA
**Epidemic PFGE types (N° isolates)**	F (6), ZO (4), R (11), N (3)	I (6), ZV (13), Y (11)	ZK (3), ZL (5), ZH (4)	ZB (4), E (5)	NA
**MLST**	ST153, ST130, ST89, ST59	ST22, ST5	ST2	ST33, ST73	NA
**N° *mec*** **A positive isolates**	6	20	12	1	‹0.001
**N° *ica*** **A positive isolates**	1	5	12	0	‹0.001
**Median sum of resistances (range)**	2 (0–6)	5 (2–8)	8 (5–8)	1 (1–4)	‹0.001

Legend:

*NA* not available.

### *In vitro* biofilm production and correlation with the presence of the *icaA* gene

**Figure 1:**
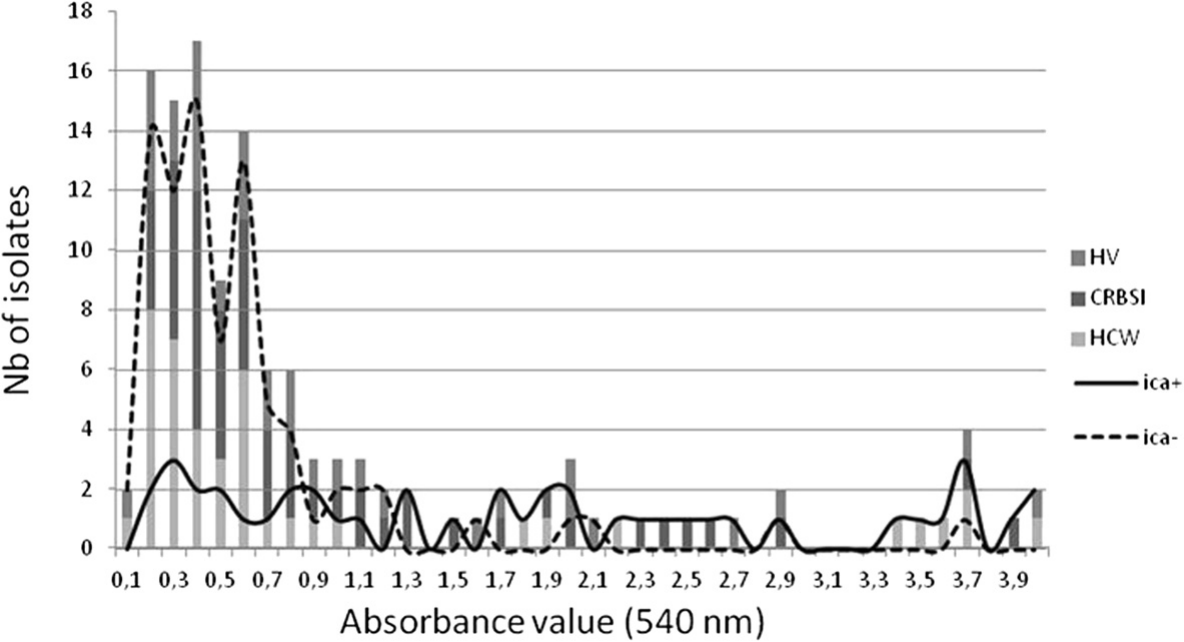
**Biofilm production and correlation with the presence of the *ica *** **A in *S*. *epidermidis *** **population among HVs (healthy volunteers), CRBSIs (catheter-related bloodstream infections) and HCWs (healthcare workers).**

One hundred and twenty-seven isolates were tested for the ability to form biofilms (one CRBSI isolate was lost). Isolates were distributed in three populations according to the importance of the biomass formation: one population with absorbance values less than 1.5, one with values between 1.5 and 3, and one population with absorbance values greater than 3 (Figure 1).

Seventy-nine of the *icaA* negative isolates (95%) presented an absorbance value that was less than 1.5, but one of the ten strong producers (absorbance value >3) was *icaA* negative, suggesting a strong but not absolute correlation between the presence of the *icaA* gene and the capacity to form a strong biofilm.

Strong biofilm producers were mainly *icaA* positive commensal isolates (six from HCWs and two from HVs), but not all *icaA* positive isolates (especially CRBSI isolates) produced strong biofilms. These isolates were evenly distributed among the three populations (Figure 1).

### Clinical data

Among the 53 CRBSI *S. epidermidis* isolates, 13 were associated with severe clinical symptoms, all were *mec* positive (*P* = 0.047), and all were resistant to a median of seven antibiotics (including beta-lactams), compared to resistance to only four antibiotics for the non-severe group (*P* = 0.03). All but one (12 out of 13) were *icaA* positive. Among the 11 isolates MLST typed, only two STs (single locus variants of one another) were found: ST2 (n = 8) and ST54 (n = 3).

## Discussion

In a previous study, we compared a set of *S. epidermidis* isolates responsible for CRBSI to a set of commensal *S. epidermidis* isolated from HVs [3]. Although almost one-quarter of CRBSI episodes were due to isolates from PFGE types that were also found to colonize the healthy students, all severe CRBSI episodes were caused by isolates belonging to “specific” PFGE types that were not found in the community. These latter isolates shared the additional characteristics of being MDR, *icaA* and *mecA* positive and belonged to MLST ST2 or ST54 (single locus variant of ST2). To understand the role of HCWs in the complex process of CRBSI, it was important to study the relatedness of *S. epidermidis* isolates from the hospital environment. The prevalence of staphylococci recovered from the HCW’s hands (82%) was similar to that reported by Cimiotti et al. [18]. We found that the *S. epidermidis* isolated from CRBSIs were significantly resistant to more antibiotics than the isolates from HCWs. For example, a higher percentage of mupirocin-resistant strains was found among isolates from CRBSIs in comparison with HCWs. The resistance of these strains could be partly due to the use of topical mupirocin calcium ointment for the eradication of nasal carriage of methicillin-resistant *S. aureus* in hospitalized patients. Conversely, resistant *S. epidermidis* isolates and especially MRSE were significantly more prevalent in HCWs than in the HV population we previously studied [3]. These findings confirm that the resistances of HCW *S. epidermidis* isolates reflect an adaptation of the HCW flora to the pressure caused by the use of antibiotics in the hospital environment, which is where colonization likely occurred. Indeed, it was previously shown that new graduate nurses acquire antibiotic-resistant staphylococci over time [18]. Conversely, Hira et al. found that characteristics of coagulase-negative staphylococci isolates from healthcare personnel changed after a period of absence from the hospital with a replacement of “hospital” strains with “community” strains [6].

Twenty-six percent (14 out of 53) of all CRBSI episodes (2006–2012) were due to isolates belonging to PFGE types that were also found on the hands of the HCWs but not among the HVs. These isolates were resistant to oxacillin and to a median of five non-beta-lactam antibiotics, but very few were *icaA* positive. They belonged to a limited number of genetic lineages (mainly ST5 and ST22), confirming that HCWs were not only colonized by hospital-adapted *S. epidermidis* clones, but they also served as a reservoir and vector for transmission to patients.

ST 73 has been previously described in isolates obtained from hospital employees or families [19]; however, in our study, five isolates belonging to the genotype PFGE type E and identified as ST73 were obtained from one HCW and 4 HVs with no contact with hospital settings.

Few CRBSI episodes (15%) were due to isolates belonging to PFGE types that were common to all the three epidemiological groups (HVs, HCWs and CRBSI patients). These isolates belonged to various genetic lineages: the vast majority was oxacillin susceptible, and all were *icaA* negative. Finally, the majority (57%) of CRBSI episodes, including all the severe CRBSI episodes caused by ST2/ST54 *icaA* and *mecA* positive MDR isolates were due to isolates belonging to PFGE types that were neither found on the skin of HV nor on the hands of HCWs.

These results should be interpreted with caution. For example, we did not check whether the nurses were newly employed at the hospital, nor did record the turnover of the nursing staff. Moreover, chlorhexidine solutions, which are used for hand hygiene, may select specific genotypes in the hospital environment, especially on the hands of HCWs [20]. Other factors could also play a role in the transmission of these particular clones. For example, environmental contamination of MRSE has been previously documented in the literature [21]. In particular, air can become contaminated with MRSE [22]: in a recent study by Botelho et al., identical clones of *S. epidermidis* were recovered from both patient and indoor air samples, and some airborne isolates displayed virulence profiles and levels of biofilm accumulation similar to those found in patient isolates [23].

*S. epidermidis* CRBSI characteristically involves biofilm formation, which is the most important factor involved in its pathogenesis [2]. In the present study, approximately 95% of *icaA* negative isolates presented only weak biofilm production while 90% of the strong biofilm producers were *icaA* positive. PIA production does not seem to be of universal importance for biofilm formation because PIA-independent biofilm formation has been demonstrated [24]. Furthermore, some isolates from biofilm-associated infections do not harbor the *ica* genes [25]. In these *ica* negative strains, biofilm formation was mediated by proteins such as the accumulation-associated protein Aap [26] or biofilm-associated protein Bap/Bhp [27]. It is now known that an insertion sequence element called IS*256* is actively involved in the modulation of biofilm formation through reversible insertion into the *ica* operon and its regulatory genes [28]. This insertion sequence occurs more frequently in MDR and *ica*-positive isolates [29]. This “phase variation” phenomenon may explain why half of our ST2/ST54 *icaA* and *mecA* positive MDR isolates were not strong *in vitro* biofilm producers.

ST2 (n = 12) isolates were exclusively found among CRBSIs and were responsible for the majority of clinically severe CRBSIs. This widespread ST, identified by Miragaia et al. [30], seems to be a highly successful strain because most of the nosocomial infections that occur worldwide are due to ST2 isolates. In a recent study, Du et al. examined 120 clinical *S. epidermidis* isolates and 204 commensal isolates in parallel (92 from HCWs and 112 from HVs) [31]. As in the present study, the ST2 isolates, almost exclusively responsible for the catheter-related infections were not recovered from HVs, were MDR and *icaA* positive; however, they were also IS*256*-positive. The authors postulated that the combination of these two genetic elements (*ica* operon and IS*256*) with antibiotic resistance leads to the success of ST2 in the hospital environment and among device-related infections.

Our study presents several limitations. First, the sample size was small (*n* = 62) and limited to a single hospital. Additionally, the sampling of HCWs (2012) was not at the exact same time compared with that for patients with CRBSI (2011–2012) because CRBSIs due to *S. epidermidis* are rare events. Furthermore, only one *S. epidermidis* strain per patient and per HCW was studied, which limits the interpretation of our results given the frequent polyclonality of *S. epidermidis* infection and colonization.

## Conclusions

*S. epidermidis* isolates from the hospital environment (CRBSIs and HCWs) were resistant to more antibiotics than *S. epidermidis* colonizing HVs. Almost one third of CRBSI episodes were due to isolates belonging to PFGE types that were also found on the hands of HCWs, but not among HVs, suggesting that HCWs serve as a reservoir for transmission to patients. However, nosocomial CRBSIs, particularly clinically severe CRBSIs were mainly due to *mecA* positive, *icaA* positive *S. epidermidis* isolates belonging to PFGE types and MLST sequence types (ST2 and ST54) that were not found on the skin of HVs or on the hands of HCWs. These findings suggest that these *S. epidermidis* genotypes, through a combination of virulence and resistance, are particularly adapted to survive in the hospital environment and cause severe catheter-related infections.

## Abbreviations

CRBSI: Catheter-related bloodstream infection; HCW: Healthcare worker; HV: Healthy volunteer; *ica*: Intercellular adhesion; ID: Identification; MALDITOF-MS: Matrix-assisted laser desorption/ionization time-of-flight mass spectrometry; MDR: Multidrug resistance; MLST: Multilocus sequence typing; MRSE: Methicillin-resistant *Staphylococcus epidermidis*; PFGE: Pulsed field gel electrophoresis; PIA: Intercellular polysaccharide adhesin; SCC*mec*: Staphylococcal cassette chromosome *mec*; ST: Sequence type.

## Competing interests

The authors declare that they have no competing interests.

## Authors’ contributions

SC and MH designed the study and drafted the manuscript. SC, BB, MH and OD analyzed and interpreted the data. AD, CNo. and CNa. performed the laboratory work, including resistance tests, molecular biology and biofilm formation study. BB helped with statistical testing and manuscript preparation. BB and MH supervised the manuscript. All authors read and approved the final manuscript.
